# Introduction

**DOI:** 10.1007/978-3-030-52873-7_1

**Published:** 2020-09-15

**Authors:** Juliane Jarke

**Affiliations:** Institute for Information Management Bremen (ifib) & Centre for Media, Communication and Information Research (ZeMKI), University of Bremen, Bremen, Germany

**Keywords:** Demographic ageing, User-centric services, Co-creation, Digital public services, e-services, Older citizens, Digital divide, Age divide, e-government

## Abstract

Increasingly public services are provided in digital form; their uptake however remains well below expectations. In particular, amongst older adults the need for public services is high while at the same time the uptake of their digital counterparts is low. One of the reasons is that many digital public services (or e-services) do not respond well to the life worlds, use contexts and use practices of its target audiences. An increasingly popular approach to design more user-centric services is co-creation with future users. It has been noted however, that in particular older adults lack the willingness (and often ability) to co-create e-services. Hence, there is an articulated need to engage older citizens in design practice, but a lack of evidence concerning successful participation approaches. This book addresses this gap by providing evidence from three co-creation projects with older adults. In order to understand the challenges and opportunities of co-creation, the book attends to the following three aspects when analysing, evaluating and comparing the three projects: (1) *Governing co-creation and sharing control*: What are the implications of different modes of governing and managing co-creation? How do (and can) specific methods facilitate the sharing of control? (2) *Sharing expertise*: How can a variety of stakeholders be engaged in meaningful ways? What are specific challenges and opportunities for sharing (lived) experiences? (3) *Enabling change*: What types of public services are most suited for co-creation and to what extend do they enable individual and/or social change?

Few societal trends are as ambivalent as demographic ageing and the increasing digitalisation of social life. On the one hand, there is an alarmist rhetoric around ageing societies in the Global North emphasising increasing demands on social security and health care systems; on the other hand, economists promise economic growth because of a growing consumer market—the so-called silver economy. Similarly, on the one hand, digitalisation and associated advances in robotics and artificial intelligence are depicted as solutions to “social problems” such as an ageing society; on the other hand, warnings are raised about a sustained “digital age divide” in which older adults remain largely excluded from many forms of social participation in this brave new digital world. The dangers of this digital age divide seem more relevant than ever in light of the current global COVID-19 pandemic which requires physical distancing and strong reliance on digital communication channels (e.g. for using public and commercial services, and for enabling social proximity).

This poses particular challenges for the digitalisation of the public sector: Increasingly services are provided in digital form; their take-up however remains well below expectations. In particular, amongst older adults the need for public services is high while at the same time the uptake of their digital counterparts is lower than expected. One of the reasons for why many older citizens do not use the internet so far, is because they do not expect to find services relevant to them (e.g. Kubicek & Lippa, 2017). In addition, many digital public services (or e-services) do not respond well enough to the life worlds, use contexts and use practices of its target audiences. One reason for the mismatch between the actual needs of older citizens and the digital services offered by public administrations—as technologies for circulating information and interacting with citizens—is based on the disparity between those designing systems and the experiences and use practices of a service’s target audience: Digital public services are based on classifications that do not correspond to the life worlds of their target user groups, but rather represent bureaucratic ways of organising and thinking. This has been called “administrative burden”—a cognitive burden inflicted on citizens to make sense of a classification not based on their own lived experience but on the oftentimes opaque work organisation of bureaucracies. It has been argued widely that the administrative burden of digital public services has to be reduced in order to ensure a higher uptake (Holgersson & Karlsson, 2014). Hence, over the past decade, the emphasis shifted from an administration centric view of “simply” digitising existing public services to a view that considers user experience first (European Commission, 2009, 2016; Kubicek, Gerhard, & Jarke, 2019).

There is hence a need for design approaches that lead to user-friendly and meaningful digital public services. In recent years, co-creation has become a buzzword that came to be considered “a cornerstone for social innovation” in the public sector (e.g. Bason, 2010; Brandsen, Steen, & Verschuere, 2018; Britton, 2017; Damodaran & Olphert, 2006; de Jong, Neulen, & Jansma, 2019; Degnegaard, 2014; Holgersson & Karlsson, 2014; Osborne, Radnor, & Strokosch, 2016; Voorberg, Bekkers, & Tummers, 2015). The interest in co-creation by public authorities and governments is occurring against a background of financial cuttings, the complexity of problems and the availability of new technologies (European Commission, 2014). It is part of increasing efforts to keep up with the digital transformation of our society while at the same time ensuring that no citizen is left behind. This is prominent in particular in two public policy fields: eGovernment^1^ and Open Government. Within eGovernment there has been a shift towards citizen-driven, citizen-centred service development to increase the uptake of services (Axelsson, Melin, & Lindgren, 2010; Holgersson, Melin, Lindgren, & Axelsson, 2018). Within Open Government, there is a call to more transparent governments and open public administrations in which a variety of civil society actors participate in “collaborative governance” (Ansell & Gash, 2008). Following collaborative governance, public agencies directly engage non-state stakeholders in a collective decision-making process throughout all stages of the policy life-cycle (Aichholzer & Strauß, 2015; Toots et al., 2017).

It has been noted however, that in particular older adults lack the willingness to participate in the design of digital public services (public e-services). One of the reasons is their modest use digital services, the other is their lack of experience in participating in co-design projects (Holgersson & Karlsson, 2014). Indeed, there is little experience in the co-creation of digital public services with older adults. Most studies or projects engaging older adults stem from participatory or user-centred design research focussing on the design of single artefacts. Those studies lack scalability when it comes to public sector innovation and its associated complex socio-technical arrangements (e.g. Oostveen & van den Besselaar, 2004; Torfing, Sørensen, & Røiseland, 2019). Hence, there is an articulated need to engage (older) citizens in the design of digital public services, but a lack of evidence concerning successful participation approaches.

This book addresses this gap by providing evidence from three co-creation projects with older adults. It is based on the EU-funded project Mobile Age^2^ in which older adults co-created map-based digital public services. The book includes in-depth accounts of two projects from Bremen, Germany and one comparative case from Zaragoza, Spain. All projects have a focus on information services concerning neighbourhoods in urban settings and relate to policy objectives such as the World Health Organisation’s Age-friendly Cities and Communities framework (GNAFCC). The projects ran over a similar length of time while following different governance structures, engagement strategies, and co-creation methods. In order to understand the challenges and opportunities of co-creating digital public services with older citizens, the book attends to the following three aspects when analysing, evaluating and comparing the three projects:

1. *Governing co-creation and sharing control*
  There is a long tradition of citizen participation in the planning, design and delivery of public services (e.g. Arnstein, 1969; Bovaird & Loeffler, 2012). However, very few studies have attended to the specific challenges of co-creating *digital* public services, and how control over design decisions and/or service delivery may be shared between governments and citizens. The difference to experiences from non-digital service co-creation is important, as there exists a tension between the local, customised and flexible use of information services on the one hand and the need for standards in public information infrastructures in order to ensure continuity and sustainability on the other (Star & Ruhleder, 1996). It is this tension that this book wants to explore further, by attending to the *implications of different modes of governing and managing co-creation as well as how specific methods facilitate the sharing control.*
2. *Sharing expertise*
  In order for co-creation projects to be successful, interventions are required that facilitate a role shift from older adults as (potential) users to co-creators. So far, our understanding of how citizens may be engaged in meaningful ways is still relatively limited (Gooch et al., 2018). Gidlund (2012) argued that there was “little systematic discussion of who users are, what they do, how they interact and what it means to use eGovernment services” (p.12). In fact, if citizens do become engaged in co-creation, their education, income, and socio-economic status are still strong, positive predictors of their civic engagement (Kavanaugh, Carroll, Rosson, Reese, & Zin, 2005). This book evaluates *how a variety of stakeholders can be engaged in meaningful ways* and identifies *specific challenges and opportunities for sharing (lived) experience to co-create digital public services for older adults*.
3. *Enabling change*
  Co-creation is based on the assumption that engaging future users in design leads to more user-friendly outputs and increases adoption. Voorberg et al. (2015) conclude in their review of 122 reports, that in the majority of cases, co-creation is considered a virtue in itself. There is too little evidence of what co-creation can actually deliver and how it may be undertaken. This book explores the *suitability of different kinds of public services for co-creation and to what extend they may ultimately enable individual and/or social change*.

In order to attend to these questions, the book is structured in the following way. The *next chapter* reviews the intricate relationship of demographic ageing and technological innovation. It argues that these are not two separate and independent phenomena, but that age is performed in relation to technology use and design (and vice versa). Chapter 10.1007/978-3-030-52873-7_3 introduces key traditions for involving citizens in the planning, design, and provision of digital public services. These include the co-production of public services, the co-design of information systems and the civic use of open (government) data. The chapter summarises and compares the different rationales for participation in these approaches, and reviews how they understand the sharing of control, the sharing of knowledge and the enabling of change. Chapter 10.1007/978-3-030-52873-7_4 introduces the Mobile Age project. It presents our framework and methodology for co-creating digital public services. The chapter introduces the problem focus of the three co-creation projects, their target audiences, resources and activities. Subsequently, each of the co-creation projects is presented in a separate chapter. Each chapter begins with a short summary of the respective co-creation project. The *two chapters about Bremen* provide in-depth accounts of the two co-creation processes. The *chapter about Zaragoza* provides a comparative case. Chapter 10.1007/978-3-030-52873-7_8 reflects on the learnings from these three co-creation projects and attends to the research questions listed above. The *book closes* with a general conclusion about how older citizens can be involved in co-creation processes in meaningful and prolific ways. This leads to a different understanding of old age and the co-creation of alternative, more inclusive socio-technical futures.

## Footnotes

1 Electronic government.

2 Mobile Age has received funding from the European Union’s Horizon 2020 research and innovation programme under grant agreement No 693319.
